# CCNE1 and survival of patients with tubo‐ovarian high‐grade serous carcinoma: An Ovarian Tumor Tissue Analysis consortium study

**DOI:** 10.1002/cncr.34582

**Published:** 2022-12-26

**Authors:** Eun‐Young Kang, Ashley Weir, Nicola S. Meagher, Kyo Farrington, Gregg S. Nelson, Prafull Ghatage, Cheng‐Han Lee, Marjorie J. Riggan, Adelyn Bolithon, Gordana Popovic, Betty Leung, Katrina Tang, Neil Lambie, Joshua Millstein, Jennifer Alsop, Michael S. Anglesio, Beyhan Ataseven, Ellen Barlow, Matthias W. Beckmann, Jessica Berger, Christiani Bisinotto, Hans Bösmüller, Jessica Boros, Alison H. Brand, Angela Brooks‐Wilson, Sara Y. Brucker, Michael E. Carney, Yovanni Casablanca, Alicia Cazorla‐Jiménez, Paul A. Cohen, Thomas P. Conrads, Linda S. Cook, Penny Coulson, Madeleine Courtney‐Brooks, Daniel W. Cramer, Philip Crowe, Julie M. Cunningham, Cezary Cybulski, Kathleen M. Darcy, Mona A. El‐Bahrawy, Esther Elishaev, Ramona Erber, Rhonda Farrell, Sian Fereday, Anna Fischer, María J. García, Simon A. Gayther, Aleksandra Gentry‐Maharaj, C. Blake Gilks, AOCS Group, Marcel Grube, Paul R. Harnett, Shariska Petersen Harrington, Philipp Harter, Arndt Hartmann, Jonathan L. Hecht, Sebastian Heikaus, Alexander Hein, Florian Heitz, Joy Hendley, Brenda Y. Hernandez, Susanna Hernando Polo, Sabine Heublein, Akira Hirasawa, Estrid Høgdall, Claus K. Høgdall, Hugo M. Horlings, David G. Huntsman, Tomasz Huzarski, Andrea Jewell, Mercedes Jimenez‐Linan, Michael E. Jones, Scott H. Kaufmann, Catherine J. Kennedy, Dineo Khabele, Felix K. F. Kommoss, Roy F. P. M. Kruitwagen, Diether Lambrechts, Nhu D. Le, Marcin Lener, Jenny Lester, Yee Leung, Anna Linder, Liselore Loverix, Jan Lubiński, Rashna Madan, G. Larry Maxwell, Francesmary Modugno, Susan L. Neuhausen, Alexander Olawaiye, Siel Olbrecht, Sandra Orsulic, José Palacios, Celeste Leigh Pearce, Malcolm C. Pike, Carmel M. Quinn, Ganendra Raj Mohan, Cristina Rodríguez‐Antona, Matthias Ruebner, Andy Ryan, Stuart G. Salfinger, Naoko Sasamoto, Joellen M. Schildkraut, Minouk J. Schoemaker, Mitul Shah, Raghwa Sharma, Yurii B. Shvetsov, Naveena Singh, Gabe S. Sonke, Linda Steele, Colin J. R. Stewart, Karin Sundfeldt, Anthony J. Swerdlow, Aline Talhouk, Adeline Tan, Sarah E. Taylor, Kathryn L. Terry, Aleksandra Tołoczko, Nadia Traficante, Koen K. Van de Vijver, Maaike A. van der Aa, Toon Van Gorp, Els Van Nieuwenhuysen, Lilian van‐Wagensveld, Ignace Vergote, Robert A. Vierkant, Chen Wang, Lynne R. Wilkens, Stacey J. Winham, Anna H. Wu, Javier Benitez, Andrew Berchuck, Francisco J. Candido dos Reis, Anna DeFazio, Peter A. Fasching, Ellen L. Goode, Marc T. Goodman, Jacek Gronwald, Beth Y. Karlan, Stefan Kommoss, Usha Menon, Hans‐Peter Sinn, Annette Staebler, James D. Brenton, David D. Bowtell, Paul D. P. Pharoah, Susan J. Ramus, Martin Köbel

**Affiliations:** ^1^ Department of Pathology and Laboratory Medicine University of Calgary Foothills Medical Center Calgary Alberta Canada; ^2^ School of Clinical Medicine UNSW Medicine and Health University of NSW Sydney Sydney New South Wales Australia; ^3^ Adult Cancer Program Lowy Cancer Research Centre University of NSW Sydney Sydney New South Wales Australia; ^4^ The Walter and Eliza Hall Institute of Medical Research Parkville Victoria Australia; ^5^ The Daffodil Centre The University of Sydney A Joint Venture With Cancer Council NSW Sydney New South Wales Australia; ^6^ Department of Oncology Division of Gynecologic Oncology Cumming School of Medicine University of Calgary Calgary Alberta Canada; ^7^ Department of Pathology and Laboratory Medicine University of Alberta Edmonton Alberta Canada; ^8^ Department of Obstetrics and Gynecology Division of Gynecologic Oncology Duke University Medical Center Durham North Carolina USA; ^9^ School of Women's and Children's Health Faculty of Medicine and Health University of NSW Sydney Sydney New South Wales Australia; ^10^ Stats Central Mark Wainwright Analytical Centre University of NSW Sydney Sydney New South Wales Australia; ^11^ Prince of Wales Clinical School UNSW Medicine and Health University of NSW Sydney Sydney New South Wales Australia; ^12^ Department of Anatomical Pathology Prince of Wales Hospital Sydney New South Wales Australia; ^13^ Canterbury Health Laboratories Christchurch New Zealand; ^14^ Division of Biostatistics Department of Population and Public Health Sciences Keck School of Medicine University of Southern California Los Angeles California USA; ^15^ Department of Oncology Centre for Cancer Genetic Epidemiology University of Cambridge Cambridge UK; ^16^ Department of Obstetrics and Gynecology University of British Columbia Vancouver British Columbia Canada; ^17^ British Columbia's Gynecological Cancer Research Team (OVCARE) University of British Columbia BC Cancer and Vancouver General Hospital Vancouver British Columbia Canada; ^18^ Department of Gynecology and Gynecologic Oncology Evangelische Kliniken Essen‐Mitte (KEM) Essen Germany; ^19^ Department of Obstetrics and Gynecology Ludwig Maximilian University Munich Munich Germany; ^20^ Gynaecological Cancer Centre Royal Hospital for Women Sydney New South Wales Australia; ^21^ Department of Gynecology and Obstetrics Comprehensive Cancer Center Erlangen‐EMN Friedrich‐Alexander University Erlangen‐Nuremberg University Hospital Erlangen Erlangen Germany; ^22^ Division of Gynecologic Oncology Department of Obstetrics, Gynecology and Reproductive Sciences University of Pittsburgh School of Medicine Pittsburgh Pennsylvania USA; ^23^ Department of Gynecology and Obstetrics Ribeirão Preto Medical School University of São Paulo Ribeirão Preto Brazil; ^24^ Institute of Pathology and Neuropathology Tuebingen University Hospital Tuebingen Germany; ^25^ Centre for Cancer Research The Westmead Institute for Medical Research University of Sydney Sydney New South Wales Australia; ^26^ Department of Gynaecological Oncology Westmead Hospital Sydney New South Wales Australia; ^27^ Discipline of Obstetrics and Gynaecology The University of Sydney Sydney New South Wales Australia; ^28^ Canada's Michael Smith Genome Sciences Centre BC Cancer Vancouver British Columbia Canada; ^29^ Department of Women's Health Tuebingen University Hospital Tuebingen Germany; ^30^ Department of Obstetrics and Gynecology John A. Burns School of Medicine University of Hawaii Honolulu Hawaii USA; ^31^ Uniformed Services of the Health Sciences Gynecologic Cancer Center of Excellence Bethesda Maryland USA; ^32^ Pathology Department Fundación Jiménez Díaz Madrid Spain; ^33^ Department of Gynaecological Oncology St John of God Subiaco Hospital Subiaco Western Australia Australia; ^34^ Division of Obstetrics and Gynaecology Medical School University of Western Australia Crawley Western Australia Australia; ^35^ Women's Health Integrated Research Center Inova Health System Falls Church Virginia USA; ^36^ Epidemiology School of Public Health University of Colorado Aurora Colorado USA; ^37^ Community Health Sciences University of Calgary Calgary Alberta Canada; ^38^ Division of Genetics and Epidemiology The Institute of Cancer Research London UK; ^39^ Obstetrics and Gynecology Epidemiology Center Department of Obstetrics and Gynecology Brigham and Women's Hospital and Harvard Medical School Boston Massachusetts USA; ^40^ Department of Epidemiology Harvard T.H. Chan School of Public Health Boston Massachusetts USA; ^41^ Department of Surgery Prince of Wales Private Hospital Randwick New South Wales Australia; ^42^ Department of Laboratory Medicine and Pathology Mayo Clinic Rochester Minnesota USA; ^43^ Department of Genetics and Pathology International Hereditary Cancer Center Pomeranian Medical University Szczecin Poland; ^44^ Gynecologic Cancer Center of Excellence Department of Gynecologic Surgery and Obstetrics Uniformed Services University of the Health Sciences Walter Reed National Military Medical Center Bethesda Maryland USA; ^45^ Henry M. Jackson Foundation for the Advancement of Military Medicine, Inc Bethesda Maryland USA; ^46^ Department of Metabolism, Digestion and Reproduction Imperial College London Hammersmith Hospital London UK; ^47^ Department of Pathology University of Pittsburgh School of Medicine Pittsburgh Pennsylvania USA; ^48^ Institute of Pathology Comprehensive Cancer Center Erlangen‐EMN Friedrich‐Alexander University Erlangen‐Nuremberg University Hospital Erlangen Erlangen Germany; ^49^ Prince of Wales Private Hospital Randwick New South Wales Australia; ^50^ Peter MacCallum Cancer Centre Melbourne Victoria Australia; ^51^ Sir Peter MacCallum Department of Oncology The University of Melbourne Parkville Victoria Australia; ^52^ Computational Oncology Group Structural Biology Programme Spanish National Cancer Research Centre (CNIO) Madrid Spain; ^53^ Center for Bioinformatics and Functional Genomics and the Cedars Sinai Genomics Core Cedars‐Sinai Medical Center Los Angeles California USA; ^54^ MRC Clinical Trials Unit Institute of Clinical Trials & Methodology University College London London UK; ^55^ Department of Pathology and Laboratory Medicine University of British Columbia Vancouver British Columbia Canada; ^56^ QIMR Berghofer Medical Research Institute Brisbane Queensland Australia; ^57^ Crown Princess Mary Cancer Centre Westmead Hospital Sydney New South Wales Australia; ^58^ Division of Gynecologic Oncology Department of Obstetrics and Gynecology The University of Kansas Medical Center Kansas City Kansas USA; ^59^ Department of Gynecology and Gynecological Oncology HSK, Dr. Horst‐Schmidt Klinik Wiesbaden Wiesbaden Germany; ^60^ Department of Pathology Beth Israel Deaconess Medical Center and Harvard Medical School Boston Massachusetts USA; ^61^ Center for Pathology Evangelische Kliniken Essen‐Mitte Essen Germany; ^62^ University of Hawaii Cancer Center Honolulu Hawaii USA; ^63^ Medical Oncology Service Hospital Universitario Funcación Alcorcón Alcorcón Spain; ^64^ Department of Obstetrics and Gynecology University Hospital Heidelberg Heidelberg Germany; ^65^ Department of Clinical Genomic Medicine Graduate School of Medicine, Dentistry and Pharmaceutical Sciences Okayama University Okayama Japan; ^66^ Department of Pathology Herlev Hospital University of Copenhagen Copenhagen Denmark; ^67^ Department of Gynaecology Rigshospitalet University of Copenhagen Copenhagen Denmark; ^68^ Division of Molecular Pathology The Netherlands Cancer Institute Amsterdam The Netherlands; ^69^ Department of Molecular Oncology BC Cancer Research Centre Vancouver British Columbia Canada; ^70^ Department of Genetics and Pathology University of Zielona Gora Zielona Gora Poland; ^71^ Department of Histopathology Addenbrooke's Hospital Cambridge UK; ^72^ Division of Oncology Research and Department of Molecular Pharmacology & Experimental Therapeutics Mayo Clinic Rochester Minnesota USA; ^73^ Division of Gynecologic Oncology Department of Obstetrics and Gynecology Washington University in St. Louis St. Louis Missouri USA; ^74^ Institute of Pathology Heidelberg University Hospital Heidelberg Germany; ^75^ Department of Obstetrics and Gynecology Maastricht University Medical Centre Maastricht The Netherlands; ^76^ GROW – School for Oncology and Reproduction Maastricht University Medical Center Maastricht The Netherlands; ^77^ Department of Human Genetics Laboratory for Translational Genetics KU Leuven Leuven Belgium; ^78^ VIB Center for Cancer Biology VIB Leuven Belgium; ^79^ Cancer Control Research BC Cancer Agency Vancouver British Columbia Canada; ^80^ International Hereditary Cancer Center Department of Genetics and Pathology Pomeranian Medical University in Szczecin Szczecin Poland; ^81^ David Geffen School of Medicine Department of Obstetrics and Gynecology University of California at Los Angeles Los Angeles California USA; ^82^ Division of Obstetrics and Gynaecology Faculty of Health and Medical Sciences University of Western Australia Crawley Western Australia Australia; ^83^ Department of Gynaecological Oncology King Edward Memorial Hospital Subiaco Western Australia Australia; ^84^ Australia New Zealand Gynaecological Oncology Group Camperdown Australia; ^85^ Department of Obstetrics and Gynecology Inst of Clinical Science, Sahlgrenska Center for Cancer Research University of Gothenburg Gothenburg Sweden; ^86^ Division of Gynecologic Oncology Department of Gynecology and Obstetrics Leuven Cancer Institute Leuven Belgium; ^87^ Department of Pathology and Laboratory Medicine The University of Kansas Medical Center Kansas City Kansas USA; ^88^ Inova Health System Women's Service Line Falls Church Virginia USA; ^89^ Department of Epidemiology University of Pittsburgh School of Public Health Pittsburgh Pennsylvania USA; ^90^ Women's Cancer Research Center Magee‐Womens Research Institute and Hillman Cancer Center Pittsburgh Pennsylvania USA; ^91^ Department of Population Sciences Beckman Research Institute of City of Hope Duarte California USA; ^92^ Department of Pathology Hospital Ramón y Cajal Instituto Ramon y Cajal de Investigación Sanitaria (IRyCIS) CIBERONC Universidad de Alcalá Madrid Spain; ^93^ Department of Epidemiology University of Michigan School of Public Health Ann Arbor Michigan USA; ^94^ Department of Epidemiology and Biostatistics Memorial Sloan‐Kettering Cancer Center New York New York USA; ^95^ Department of Population Health and Public Health Sciences Keck School of Medicine University of Southern California Norris Comprehensive Cancer Center Los Angeles California USA; ^96^ The Health Precincts Biobank UNSW Biospecimen Services Mark Wainwright Analytical Centre University of NSW Sydney Sydney New South Wales Australia; ^97^ Hereditary Endocrine Cancer Group Spanish National Cancer Research Center (CNIO) Madrid Spain; ^98^ Centre for Biomedical Network Research on Rare Diseases (CIBERER) Instituto de Salud Carlos III Madrid Spain; ^99^ Women's Cancer Institute for Women's Health University College London London UK; ^100^ Department of Epidemiology Rollins School of Public Health Emory University Atlanta Georgia USA; ^101^ Tissue Pathology and Diagnostic Oncology Westmead Hospital Sydney New South Wales Australia; ^102^ Department of Pathology Barts Health National Health Service Trust London UK; ^103^ Department of Medical Oncology The Netherlands Cancer Institute ‐ Antoni van Leeuwenhoek Hospital Amsterdam The Netherlands; ^104^ School for Women's and Infants' Health University of Western Australia Perth Australia; ^105^ Division of Breast Cancer Research The Institute of Cancer Research London UK; ^106^ Gynaepath WA Clinipath (Sonic Healthcare) Osbourne Park Australia; ^107^ Department of Genetics and Pathology Pomeranian Medical University Szczecin Poland; ^108^ Department of Pathology Ghent University Hospital Cancer Research Institute Ghent (CRIG) Ghent Belgium; ^109^ Department of Pathology Antwerp University Hospital Antwerp Belgium; ^110^ Department of Research Netherlands Comprehensive Cancer Organization (IKNL) Utrecht The Netherlands; ^111^ Department of Quantitative Health Sciences Division of Clinical Trials and Biostatistics Mayo Clinic Rochester Minnesota USA; ^112^ Department of Quantitative Health Sciences Division of Computational Biology Mayo Clinic Rochester Minnesota USA; ^113^ Human Genetics Group Spanish National Cancer Research Centre (CNIO) Madrid Spain; ^114^ Department of Quantitative Health Sciences Division of Epidemiology Mayo Clinic Rochester Minnesota USA; ^115^ Cancer Prevention and Control Program Cedars‐Sinai Cancer Cedars‐Sinai Medical Center Los Angeles California USA; ^116^ Cancer Research UK Cambridge Institute University of Cambridge Cambridge UK; ^117^ Department of Public Health and Primary Care Centre for Cancer Genetic Epidemiology University of Cambridge Cambridge UK

**Keywords:** CCNE1 amplification, cyclin E1 expression, high‐grade serous carcinoma, ovarian cancer, prognosis

## Abstract

**Background:**

Cyclin E1 (CCNE1) is a potential predictive marker and therapeutic target in tubo‐ovarian high‐grade serous carcinoma (HGSC). Smaller studies have revealed unfavorable associations for *CCNE1* amplification and CCNE1 overexpression with survival, but to date no large‐scale, histotype‐specific validation has been performed. The hypothesis was that high‐level amplification of *CCNE1* and CCNE1 overexpression, as well as a combination of the two, are linked to shorter overall survival in HGSC.

**Methods:**

Within the Ovarian Tumor Tissue Analysis consortium, amplification status and protein level in 3029 HGSC cases and mRNA expression in 2419 samples were investigated.

**Results:**

High‐level amplification (>8 copies by chromogenic *in situ* hybridization) was found in 8.6% of HGSC and overexpression (>60% with at least 5% demonstrating strong intensity by immunohistochemistry) was found in 22.4%. *CCNE1* high‐level amplification and overexpression both were linked to shorter overall survival in multivariate survival analysis adjusted for age and stage, with hazard stratification by study (hazard ratio [HR], 1.26; 95% CI, 1.08‐1.47, *p* = .034, and HR, 1.18; 95% CI, 1.05‐1.32, *p* = .015, respectively). This was also true for cases with combined high‐level amplification/overexpression (HR, 1.26; 95% CI, 1.09‐1.47, *p* = .033). *CCNE1* mRNA expression was not associated with overall survival (HR, 1.00 per 1‐SD increase; 95% CI, 0.94‐1.06; *p* = .58). *CCNE1* high‐level amplification is mutually exclusive with the presence of germline *BRCA1/2* pathogenic variants and shows an inverse association to RB1 loss.

**Conclusion:**

This study provides large‐scale validation that *CCNE1* high‐level amplification is associated with shorter survival, supporting its utility as a prognostic biomarker in HGSC.

## Abstract 2

Multi‐institutional validation of the prognostic value of cyclin E1 high‐level amplification and overexpression in 3029 tubo‐ovarian high‐grade serous carcinoma cases support its value as a prognostic biomarker in this disease.

## INTRODUCTION

Cyclin E1 (CCNE1) is a potential predictive marker and therapeutic target in tubo‐ovarian high‐grade serous carcinoma (HGSC).^1^, ^2^ CCNE1 has three main functions in cell‐cycle progression.^3^ First, it is involved in the formation of prereplication minichromosome maintenance protein complexes, which bind origins of DNA replications as cells reenter G1‐ from G0‐phase of the cell cycle. Second, by forming a complex, it activates the cyclin‐dependent kinase CDK2 to phosphorylate several targets including RB1, which subsequently abandons its inhibition of E2F transcription factors and initiates the transition from G1 to S phase.^3^ CDK2 inhibition by cyclin‐dependent kinase inhibitors 1 (CDKN1a/p21) is dependent on normal TP53 function. Third, the CDK2/CCNE1 complex promotes centrosome duplication.^3^, ^4^ Normal CCNE1 protein levels are tightly regulated, peaking in late G1 and decreasing as cells progress through S phase.^5^ In neoplasia, CCNE1 protein overexpression is uncoupled from the cell cycle.^6^ Constitutive overexpression of CCNE1, but not of CCND1 or CCNA, induces chromosomal instability and a modest degree of polyploidy.^6^ The mechanisms by which CCNE1 causes chromosomal instability are not entirely understood, but it has been suggested that cells with deregulated CCNE1 prematurely enter S phase with inadequate nucleotide pools, causing replication stress with faulty replication forks engendering DNA double‐stranded breaks.^7^, ^8^

In ovarian carcinoma, *CCNE1* amplification has been associated with resistance to platinum‐based chemotherapy and shorter overall survival.^9^, ^10^ However, the cutoff for amplification varies among studies. Larger studies such as The Cancer Genome Atlas project reported only a suggestive trend toward shorter overall survival (*p* = .0718) and another study of 179 HGSC showed evidence for a significant association only with progression‐free survival.^11^, ^12^ Amplification of the chromosomal region 19q12 containing the *CCNE1* gene is common (∼20%) in HGSC, which across all tumor sites ranks third in frequency after endometrial carcinosarcoma and urothelial carcinoma.^13^ *CCNE1* amplification is inversely associated with germline pathogenic *BRCA1/2* variants, which becomes mutually exclusive for high‐level amplifications (defined by >8 copies).^14^, ^15^ *CCNE1* high‐level amplified HGSC require proficient homologous recombination, including BRCA1/2 function to maintain cell viability.^14^, ^15^ *CCNE1* high‐level amplification is the lead alteration for both the copy number signature 6 and the fold‐back inversion mutation signature, which characterize homologous recombination‐proficient HGSC.^16^, ^17^ Patients with HGSC and homologous recombination‐proficient tumors do not respond well to chemotherapy or poly (ADP‐ribose) polymerase (PARP) inhibitors. For example, PARP maintenance therapy for patients with homologous recombination‐proficient HGSC and partial chemotherapy response resulted in a median progression‐free survival of 8.3 months compared with 21.9 months for patients with homologous recombination‐deficient HGSC.^18^

Although no association of *CCNE1* mRNA expression with survival in HGSC has been observed,^15^, ^19^ CCNE1 protein overexpression has been associated with unfavorable outcomes in ovarian carcinomas, albeit only in studies conducted before the era of histotype‐specific analysis.^20^, ^21^, ^22^ Two recent studies suggested that the combination of *CCNE1* amplifications and CCNE1 overexpression is associated with shorter survival.^15^, ^23^ We recently validated a *CCNE1* chromogenic *in situ* hybridization (CISH) assay orthogonally against other copy number assays to be applicable on tissue microarrays and refined the cutoff for immunohistochemistry to detect *CCNE1* high‐level gene amplifications.^15^

Here, our objectives were to validate previously reported associations of CCNE1 alterations with overall survival; assess correlations between *CCNE1* high‐level gene amplifications, *CCNE1* mRNA, and CCNE1 protein expression; and explore associations with selected biomarkers in a large cohort of HGSC samples from the international Ovarian Tumor Tissue Analysis (OTTA) consortium.

## METHODS

### Study cohort

Twenty studies from the OTTA consortium participated in the current study.^24^ Each study enrolling patients received local ethics review board approval (Table S1). Tissue microarrays were constructed from formalin‐fixed paraffin‐embedded tumor specimens obtained from debulking surgery representing each tumor with one to three cores, 0.6 to 1.0 mm in size. For both CISH and immunohistochemistry (IHC), data were successfully obtained in 3029 samples from patients with HGSC. Clinical covariates, time to follow up, and status were centrally standardized. Cases were collected during the pre‐PARP inhibitor era. Platinum‐based chemotherapy was given in the majority as adjuvant therapy after primary debulking surgery or as neoadjuvant chemotherapy. Information on specific drugs was not collected. Previously generated IHC data within the OTTA consortium for TP53, CDKN2A, and RB1 were used.^25^, ^26^, ^27^

### CCNE1 DNA CISH

A previously published in‐house CISH protocol using a commercial digoxigenin (DIG)‐labeled *CCNE1* DNA probe (Empire Genomics, Buffalo, NY, USA) was used.^15^ Deparaffinized 4‐μm tissue microarray sections were pretreated with proteinase K (3 min) and citrate‐based antigen retrieval buffer at 80°C (1 h) followed by pepsin (45 sec), and then dehydrated and air‐dried. Hybridization with the DIG‐labeled *CCNE1* probe was performed at 37°C for 16 to 18 hours in HybEZ II (Advanced Cell Diagnostics, Minneapolis, MN, USA). A levamisole solution was used (15 min) to remove endogenous alkaline phosphatase activity, followed by a blocking solution (30 min) of 10% normal sheep serum, 2% bovine serum albumin, and 0.05% Tween‐20. An alkaline phosphatase‐conjugated sheep anti‐DIG antibody (dilution 1:800; Roche, Basel, Switzerland) was incubated for 2 h. An alkaline phosphatase substrate was applied, and the reaction was stopped with 50 mM Tris, 150 mM NaCl, and 10 mM KCl buffer when slides reached the desired intensity of staining. Counterstaining was performed with hematoxylin.

### CCNE1 immunohistochemistry

Four‐μm sections from tissue microarrays were deparaffinized, rehydrated, and subjected to heat‐induced epitope retrieval on the DAKO Omnis platform (Agilent Technologies, Santa Clara, CA, USA), followed by incubation with the CCNE1 antibody (1:600, clone EP126, Cell Marque, Rocklin, CA, USA; 30‐10R‐30) at room temperature and in the EnVision FLEX (Agilent Technologies). The reaction was visualized using 3,3‐diaminobenzidine tetrahydrochloride for 10 min and counterstained with hematoxylin.

### CCNE1 CISH and IHC scoring

The *CCNE1* CISH assay was previously orthogonally validated to detect *CCNE1* high‐level amplifications (presence of clusters >8 copies) against a digital polymerase chain reaction and nCounter Cancer CN Assay.^15^ CCNE1 protein expression showed a wide and relatively even distribution from 5% to 90% of positive tumor cells, but previous receiver operating characteristic curve analysis established an optimal cutoff for IHC to detect high‐level amplification at >60% overall staining cells with at least 5% showing strong intensity.^15^ The interpretation of tissue microarrays was performed by three pathologists. Training was provided on a set of 90 HGSC cases guided by illustrated examples. Subsequently, interobserver reproducibility was tested on 415 cases. The interobserver observer reproducibility for paired observers achieved a Cohen's kappa of 0.48, 0.55, and 0.77 for CISH and 0.65, 0.75, and 0.85 for IHC using a binary categorization. Subsequently, examples of discordant cases were discussed at a multiheaded microscope to further align interpretational thresholds and equivocal categories were allowed both for CISH and IHC.^15^ Each observer subsequently scored approximately one‐third of the cases by using the following criteria for CISH: score 0, no clusters = negative for high‐level amplification (CCNE1^nonamp^); score 1, equivocal favor negative; score 2, equivocal favor high; and score 3, nuclear clusters of CISH signal = high‐level amplification (CCNE1^amp^); and for IHC score 0, <60% positive tumor cells (CCNE1^lo^); score 1, equivocal favor low; score 2, equivocal favor high; and score 3, ≥60% positive tumor cells with at least 5% strongly staining cells (CCNE1^hi^).

### CCNE1 mRNA expression by NanoString

Formalin‐fixed paraffin‐embedded tumor specimens (*n* = 2419) with partial overlap to the previous specimens (1612/3029) were obtained from additional cores or sections.^28^ RNA extraction methods, assay run parameters, data processing, and control/reference samples were as previously described.^29^ *CCNE1* mRNA expression was assessed using the NanoString nCounter technology; the *CCNE1* target sequence was CCTCCAGACACCAGTGCGTGCTCCCGATGCTGCTATGGAAGGTGCTACTTGACCTAAGGGACTCCCACAACAACAAAAGCTTGAAGCTGTGGAGGGCCAC, and *CCNE1* mRNA data were normalized against housekeeping genes.^29^ Quality assurance of the assay was previously performed with high duplicate sample correlation.^19^, ^30^

### Statistical analyses

Correlations between *CCNE1* mRNA, gene amplification (ISH), and protein (IHC) overexpression were measured using Pearson correlation coefficients. Chi‐square proportions testing was undertaken to evaluate clinical and molecular variables across CCNE1 combinations. Univariate and multivariate survival analyses of *CCNE1* profiles were performed. Overall survival (death from any cause) was the primary end point. Potential survival bias introduced by the time between diagnosis and study enrollment was moderated by left truncation. Deaths potentially unrelated to HGSC were right censored at 10 years from diagnosis. The Kaplan–Meier method, alongside log‐rank testing, was used to assess overall survival by *CCNE1* profile. Multivariate Cox proportional hazards regression modeling, stratified by the OTTA study, complemented this analysis through estimation of hazard ratios (HRs) with 95% CIs. The covariates, age, stage, completeness of surgical cytoreduction (residual disease vs. no residual disease [sensitivity analysis]), and *CCNE1* profiles were adjusted for, and different baseline hazards of OTTA studies were stratified, in multivariate models. Scaled Schoenfeld residuals assessed the assumption of proportional hazards. All statistical analyses were performed using RStudio v1.1.463 or GraphPad Prism v7.02. Statistical significance was defined by *p* < .05.

## RESULTS

### Prevalence of CCNE1 high‐level amplification and association with overall survival

**FIGURE 1:**
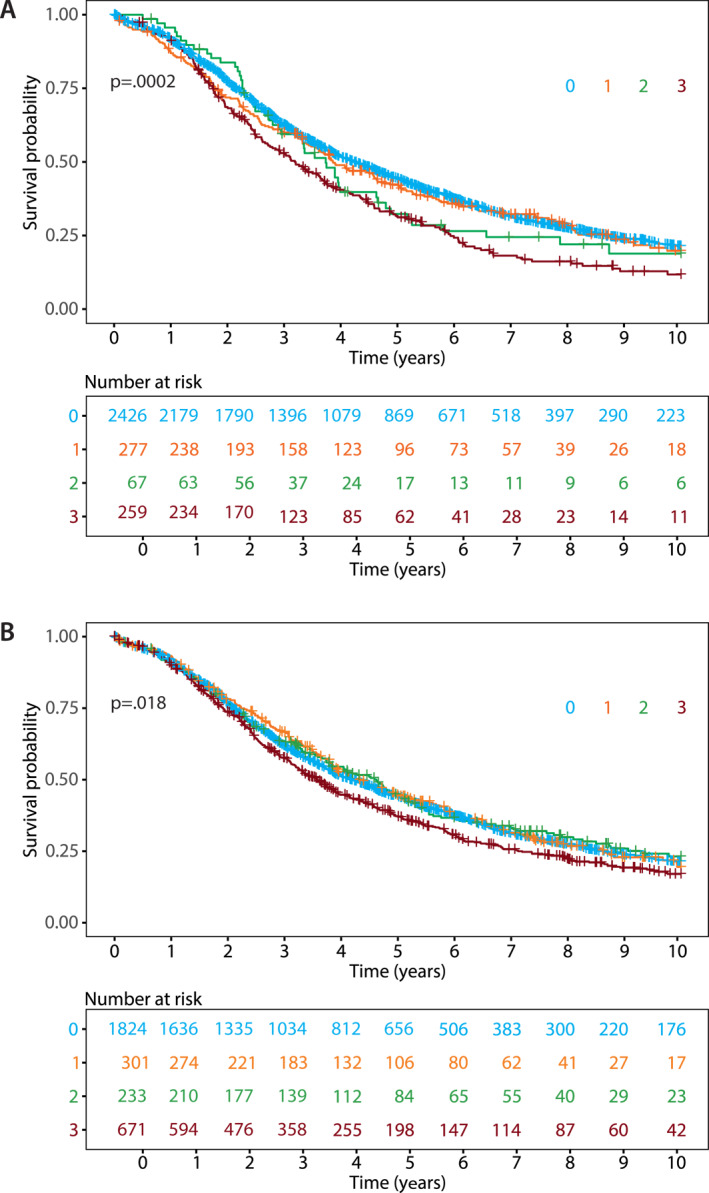
Kaplan–Meier overall survival analyses for (A) CISH score levels and (B) IHC score levels. CISH indicates chromogenic *in situ* hybridization; IHC, immunohistochemistry

*CCNE1* CISH showed high‐level amplification (score 3) in 259/3029 (8.6%) cases and 2426/3029 (80.2%) demonstrated no evidence of amplification (score 0). The remainder were equivocal with 67/3029 (2.2%) favored high‐level amplification (score 2), and 277/3029 (9.1%) not favored (score 1). Kaplan–Meier survival analysis showed a significantly different overall survival among the groups (log‐rank *p* = .00016; Figure 1A). In multivariate analysis adjusted for age and stage and stratified for the OTTA study, *CCNE1* high‐level amplified HGSC showed an HR of 1.26 (95% CI, 1.08–1.47) compared with the reference group with no evidence of amplification (Table 1). Data on the completeness of surgical cytoreduction were available for a subset of cases (66.9%) and, within this group, a sensitivity analysis adjusted for age, stage, completeness of surgical cytoreduction, and stratified for the OTTA study, resulted in the same HR of 1.27 (95% CI, 1.06–1.52; Table S2).

**TABLE 1:** Multivariate association between the expression and amplification of CCNE1 and overall survival in high‐grade serous ovarian carcinoma (*n* = 3029)

CCNE1 profile	No.^a^	5‐year survival (% ± SE)	Hazard ratio (95% CI)^b^	*p*
*CCNE1* CISH score 0	2426	41.9 ± 1.1	Referent	.034^c^
*CCNE1* CISH score 1	277	40.2 ± 3.1	0.98 (0.84–1.14)	
*CCNE1* CISH score 2	67	32.1 ± 6.2	0.97 (0.72–1.31)	
*CCNE1* CISH score 3	259	29.5 ± 3.0	1.26 (1.08–1.47)*	
CCNE1 IHC score 0	1824	41.6 ± 1.2	Referent	.015^c^
CCNE1 IHC score 1	301	43.0 ± 3.0	0.99 (0.85–1.16)	
CCNE1 IHC score 2	233	42.8 ± 3.4	0.92 (0.78–1.10)	
CCNE1 IHC score 3	671	35.4 ± 2.0	1.18 (1.05–1.32)*	
CCNE1^nonamp_lo^	2064	41.9 ± 1.2	Referent	.033^c^
CCNE1^nonamp_hi^	639	41.0 ± 2.1	1.04 (0.93–1.16)	
CCNE1^amp_lo^	61	37.8 ± 6.6	0.97 (0.71–1.34)	
CCNE1^amp_high^	265	28.3 ± 3.0	1.26 (1.09–1.47)^c^	

Abbreviations: CCNE1, cyclin E1; CCNE1^amp^, CCNE1 high‐level amplification; CCNE1^hi^, CCNE1 protein overexpression by immunohistochemistry; CCNE1^lo^, negative for CCNE1 protein overexpression by immunohistochemistry; CCNE1^nonamp^, negative for CCNE1 high‐level amplification; CISH, chromogenic in situ hybridization; HGSC, high‐grade serous ovarian carcinoma; HR, hazard ratio; IHC, immunohistochemistry; OS, overall survival.

^a^ The same cohort was assessed in univariate survival analysis.

^b^ HR adjusted for patient age and stage, with stratification by the Ovarian Tissue Tumor Analysis study; Cox proportional regression modeling was used to calculate *p* values and define significance.

^c^ Statistically significant values.

*p < .05.

### Prevalence of CCNE1 protein overexpression and association with overall survival

CCNE1 IHC showed overexpression (score 3) in 671/3029 (22.2%) cases and 1824/3029 (60.2%) had low CCNE1 protein levels (score 0) (Table 1). The remainder were equivocal, with 233/3029 (7.7%) favored to express high protein levels (score 2) and 301/3029 (9.9%) favored to express low levels (score 1). Kaplan–Meier survival analysis showed a significantly different survival between the groups (log‐rank *p* = .021; Figure 1B). In multivariate analysis adjusted for age and stage, and stratified for OTTA study, CCNE1 high protein level HGSC showed an HR of 1.18 (95% CI, 1.05–1.32) compared with the group with low CCNE1 protein levels (Table 1). In a sensitivity multivariate analysis adjusted for age, stage, completeness of surgical cytoreduction, and stratified for OTTA study, a similar HR of 1.20 (95% CI, 1.05–1.37) was obtained (Table S2).

### Associations of combined CCNE1 high‐level amplification and protein level with overall survival

**FIGURE 2:**
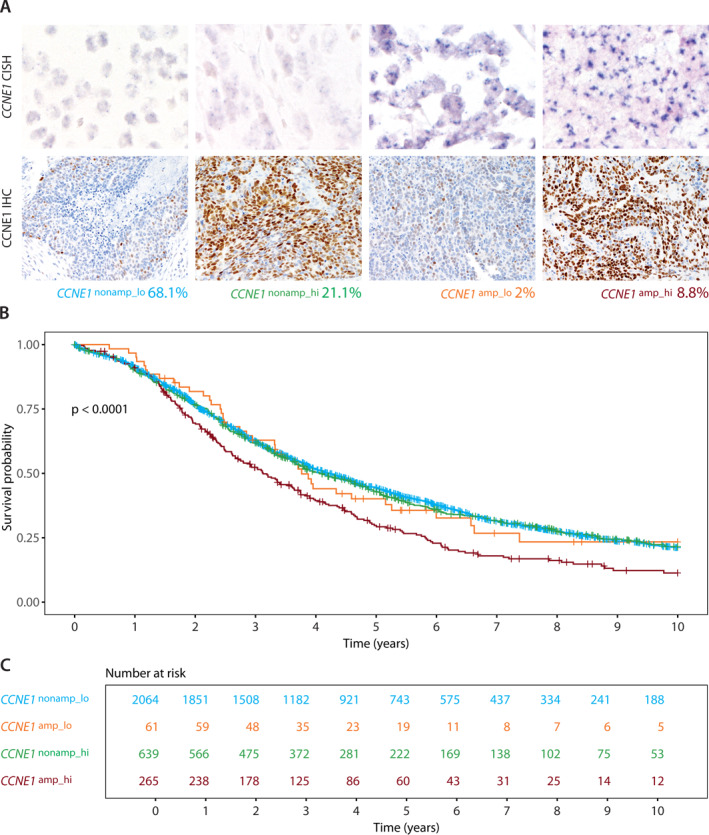
(A) *CCNE1* DNA CISH and IHC combinations resulting in four groups: CCNE1^nonamp_lo^ CISH showing no high‐level amplification and IHC <60% positive tumor cell nuclei, CCNE1^nonammp_hi^ CISH showing no high‐level amplification and IHC >60% positive and >5% strongly staining tumor cell nuclei, CCNE1^amp_lo^ CISH showing high‐level amplification and IHC <60% positive tumor cell nuclei, CCNE1^amp_hi^ CISH showing high‐level amplification, and IHC >60% positive and >5% strongly staining tumor cell nuclei. (B) Kaplan–Meier overall survival analysis for four combined CISH/IHC groups. (C) Risk table indicating the number of patients within the cohort that are at risk of death, observed at a yearly. CISH, chromogenic *in situ* hybridization; IHC, immunohistochemistry

After binarization of CISH and IHC scores into scores 0/1 versus scores 2/3, 265/326 (81.3%) of high‐level amplified cases showed high CCNE1 protein levels and, conversely, 2064/2703 (76.4%) of non–high‐level amplified cases showed low CCNE1 protein levels. We then combined CCNE1 CISH and IHC into four groups (Figure 2, Table S3): first, negative for *CCNE1* high‐level amplification with low CCNE1 protein expression (CCNE1^nonamp_lo^) comprising 68.1% (2064/3029) of the cases; second, negative for *CCNE1* high‐level amplification but with CCNE1 protein overexpression (CCNE1^nonamp_hi^) comprising 21.1% (639/3029); third, *CCNE1* high‐level amplification but low CCNE1 protein expression (CCNE1^amp_lo^) comprising 2.0% (61/3029); and fourth, *CCNE1* high‐level amplification with CCNE1 protein overexpression (CCNE1^amp_hi^) comprising 8.8% (265/3029) of cases (Table 1). Kaplan–Meier survival analysis showed a significantly different overall survival among the groups (log‐rank *p* < .0001, Figure 2). Patients in the CCNE1^amp_hi^ group had a 5‐year survival rate of 28.3% compared with 41.9% in the CCNE1^nonamp_lo^ group (Table 1). This difference remained significant in multivariate modeling, following adjustment for age and stage and stratified for the OTTA study. The CCNE1^amp_high^ group had a higher risk of death (HR, 1.26; 95% CI, 1.09–1.47) compared with the reference CCNE1^nonamp_lo^ group (Table 1). In a sensitivity analysis adjusted for age, stage, completeness of surgical cytoreduction, and stratified for the OTTA study, the hazard ratio for the CCNE1^amp_high^ group compared with the reference CCNE1^nonamp_lo^ group was 1.20 (95% CI, 1.00–1.43; Table S2).

### Associations of combined CCNE1 high‐level amplification and protein expression with clinical parameters and biomarkers in HGSC

The univariate associations of the combined groups with clinicopathological parameters are shown in Table 2. Patients diagnosed with *CCNE1* high‐level amplified HGSC were older, with a trend toward a higher likelihood of residual disease after debulking surgery. No associations were observed for stage (International Federation of Gynecology and Obstetrics I, II [locoregional] compared with International Federation of Gynecology and Obstetrics III/IV [distant]) or the timing of the primary chemotherapy regimen (adjuvant vs. neoadjuvant; Table S4). For subsets with available data, the four groups showed significant associations with TP53 IHC (available data for 65.9% of cases), *BRCA1/2* germline variant (available data for 31.5% of cases), CDKN2A IHC (available data for 64.2% of cases), and RB1 IHC status (available data for 71.1% of cases; Table 3). Normal TP53 IHC was most prevalent in the CCNE1^nonamp_lo^ group. However, the abnormal TP53 IHC patterns, which are surrogates for the functional groups of *TP53* mutations,^31^ were not different. Germline *BRCA1/2* mutations were rarely present in *CCNE1* high‐level amplified HGSC. Only two HGSC cases had protein‐truncating deleterious *BRCA2* variants and both cases had a *CCNE1* CISH score of 2 (equivocal favor high). The CCNE1^amp_high^ group had the highest frequency of CDKN2A block expression, a surrogate for RB pathway activation, but there were no cases with complete absence of CDKN2A expression, a surrogate for a deleterious deletion of *CDKN2A*. *CCNE1* high‐level amplification was inversely associated with loss of RB1.

**TABLE 2:** Clinicopathological parameters by combined CCNE1 protein and amplification status (*n* = 3029)

Clinicopathological variable	CCNE1 profile				*p* ^a^	Total
	CCNE1^nonamp_lo^	CCNE1^nonamp_hi^	CCNE1^amp_lo^	CCNE1^amp_hi^		
Number of cases, *n* (%)^b^	2064 (68.1)	639 (21.1)	61 (2.0)	265 (8.8)		3029 (100.0)
Age at diagnosis, years						
Mean ± SD	60.9 ± 11.4	61.7 ± 10.9	65.0 ± 9.11	65.0 ± 9.8		61.5 ± 11.2
Median	61	62	66	65		62
Range	21–93	30–92	40–84	38–91		21–93
Stage, *n* (%)^c^					.3848	
FIGO I, II (locoregional)	350 (17.0)	124 (19.4)	9 (14.8)	41 (15.5)		525 (17.3)
FIGO III, IV (distant)	1714 (83.0)	515 (80.6)	52 (85.2)	224 (84.5)		2527 (82.7)
Completeness of survival cytoreduction					.0563^d^	
No residual disease, *n* (%)^c^	555 (40.7)	200 (44.5)	10 (34.5)	61 (33.2)		826 (40.8)
Residual disease present, *n* (%)^c^	809 (59.3)	249 (55.5)	19 (65.5)	123 (66.9)		1200 (59.2)
Unknown, *n* ^c^	700	190	32	81		1003

Abbreviations: CCNE1, cyclin E1; CCNE1^amp^, CCNE1 high‐level amplification; CCNE1^hi^, CCNE1 protein overexpression by immunohistochemistry; CCNE1^lo^, negative for CCNE1 protein overexpression by immunohistochemistry; CCNE1^nonamp^, negative for CCNE1 high‐level amplification; FIGO, International Federation of Gynecology and Obstetrics.

^a^ Chi‐square testing was used to calculate *p* values. Statistically significant values shown; *p* < .05.

^b^ The proportion of cases in each score stratum is given as a percentage of the total patients examined.

^c^ The proportion of cases is given as a percentage of the total cases within each score stratum.

^d^ Chi‐square testing to compare the proportions of cases with absent vs. present residual disease status. This does not include cases in which residual disease status was unknown.

**TABLE 3:** Univariable associations with selected biomarkers by combined CCNE1 protein and amplification status (*n* = 3029)

	Status	CCNE1 profile^a^				*p* ^c^	Total^d^
Molecular marker^b^		CCNE1^nonamp_lo^	CCNE1^nonamp_hi^	CCNE1^amp_lo^	CCNE1^amp_hi^		
TP53	Abnormal	1202 (89.9)	413 (94.3)	42 (100.0)	172 (95.6)	.0008^e^	1829 (91.6)
	Normal	135 (10.1)	25 (5.7)	0 (0.0)	8 (4.4)		168 (8.4)
	Unknown	727	201	19	85		1032
Abnormal TP53 IHC patterns^f^	Abnormal OE	830 (69.1)	296 (71.7)	31 (73.8)	120 (69.8)	.8706	1277 (69.8)
	Abnormal CA	311 (25.9)	98 (23.7)	10 (23.8)	46 (26.7)		465 (25.4)
	Abnormal CY	61 (5.1)	19 (4.6)	1 (2.4)	6 (3.5)		87 (4.8)
*BRCA1/2* germline pathogenic variant	Present	111 (16.9)	33 (16.5)	0 (0.0)	2 (2.9)	.0020^e^	146 (15.3)
	Absent	546 (83.1)	167 (83.5)	28 (100.0)	67 (97.1)		808 (84.7)
	Unknown	1407	439	33	196		2075
CDKN2A	Normal	630 (48.2)	125 (29.7)	12 (30.8)	33 (18.6)	<.0001^e^	800 (41.2)
	Abnormal block positive	591 (45.2)	288 (68.4)	24 (61.5)	144 (81.4)		1047 (53.9)
	Abnormal complete absence	86 (6.6)	8 (1.9)	3 (7.7)	0 (0.0)		97 (5.0)
	Unknown	757	218	22	88		1085
RB1	Normal (retained)	1153 (81.1)	402 (83.6)	44 (97.8)	187 (91.2)	.0001^e^	1786 (83.0)
	Abnormal (loss)	269 (18.9)	79 (16.4)	1 (2.2)	18 (8.8)		367 (17.0)
	Unknown	642	158	16	60		876
Total^c^		2064 (68.1)	639 (21.1)	61 (2.0)	265 (8.8)		3029 (100.0)

Abbreviations: CA, complete absence; CCNE1, cyclin E1; CCNE1^amp^, CCNE1 high‐level amplification; CCNE1^hi^, CCNE1 protein overexpression by immunohistochemistry; CCNE1^lo^, negative for CCNE1 protein overexpression by immunohistochemistry; CCNE1^nonamp^, negative for CCNE1 high‐level amplification; CY, cytoplasmic; IHC, immunohistochemistry; OE, overexpression.

^a^ CCNE1 profile amplification is defined by chromogenic *in situ* hybridization and, and protein expression is defined by immunohistochemistry.

^b^ The proportion of cases with a particular molecular marker status is given as a percentage of the total patients examined in each CCNE1 profile. This does not include cases where mutational status was unknown.

^c^ The proportion of cases in each CCNE1 profile is given as a percentage of the total patients examined. This does not include cases where mutational status was unknown.

^d^ Chi‐square testing was used to calculate *p* values. This does not include cases where mutational status was unknown.

^e^ Statistically significant values; *p* < .05.

^f^ TP53 type of abnormal mutation‐type immunohistochemical pattern: OE, CA, and CY.

### CCNE1 mRNA expression by NanoString in HGSC

**FIGURE 3:**
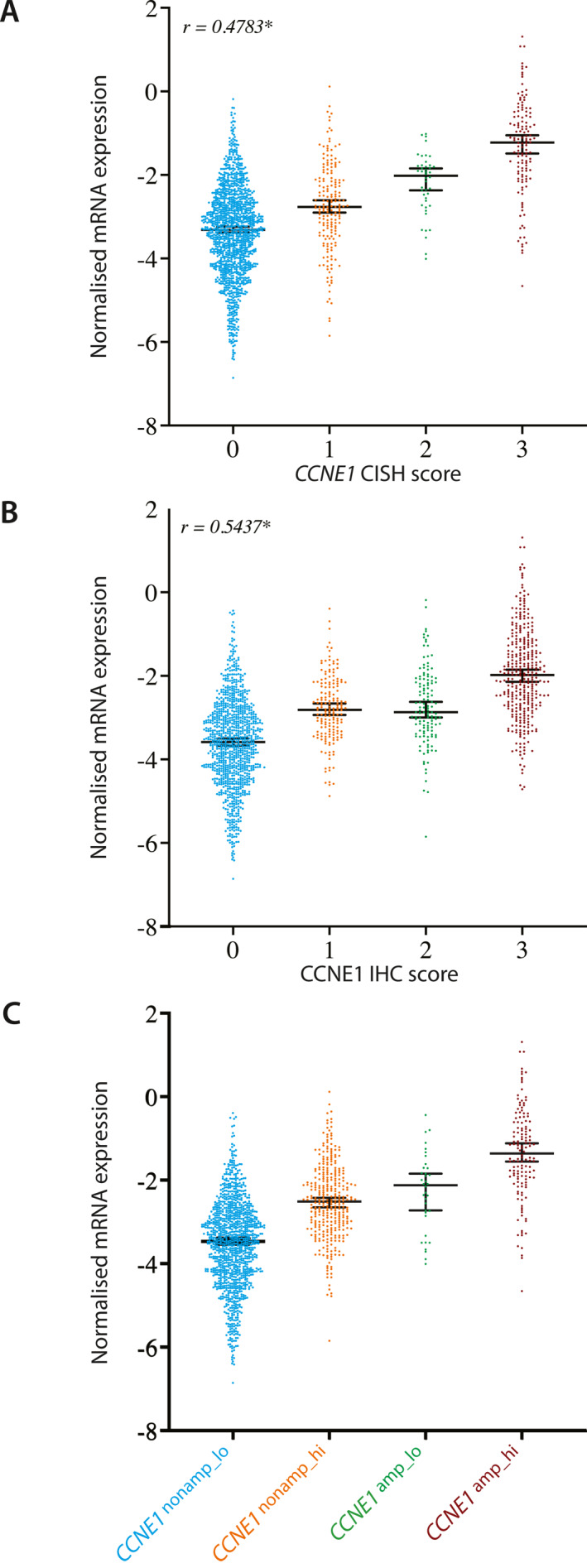
(A) Correlation of CCNE1 DNA CISH score with normalized mRNA expression. (B) Correlation of CCNE1 protein IHC score with normalized RNA expression. (C) Association of four combined CISH/IHC groups with normalized RNA expression. Pearson's correlation analysis given by *r*. *p < .05. CCNE1 indicates cyclin E1; CISH, chromogenic *in situ* hybridization; IHC, immunohistochemistry

For 1612/3029 overlapping cases with *CCNE1* mRNA expression, there was moderate correlation between *CCNE1* mRNA expression and CISH scores (*r* = 0.478) and CCNE1 IHC scores (*r* = 0.544; Figure 3). *CCNE1* mRNA expression was also different across the four combined groups, with the highest level observed in CCNE1^amp_high^, followed by CCNE1^amp_lo^ and CCNE1^nonamp_hi^ (Figure 3). Lastly, we evaluated the associations of *CCNE1* mRNA expression with overall survival in 2419 HGSC cases. The clinicopathological characteristics of these cases are shown in Table S5. When considering a 1‐SD increase in *CCNE1* mRNA expression score, there was no association with overall survival (HR, 1.00; 95% CI, 0.94–1.06, *p* = .96; Table S6). This also was the case when using a cutoff at the top 10% versus the remainder (HR, 1.06; 95% CI, 0.88–1.27, *p* = .53; Table S6).

## DISCUSSION

In this study, we validate the association of combined *CCNE1* high‐level gene amplification and CCNE1 protein overexpression with overall survival in a large cohort of patients with HGSC from the OTTA consortium. Our results demonstrate that assessing *CCNE1* at a DNA copy‐number level and protein level is a more robust determinant of prognosis than mRNA expression. We also confirm that *CCNE1* high‐level amplification is essentially mutually exclusive with pathogenic *BRCA1/2* germline alterations and associated with biomarker changes in the RB1 pathway.

For the association of CCNE1 protein expression with survival, the genomic context seems to matter. The fairly large group of CCNE1^nonamp_hi^ shows a similar survival compared with the CCNE1^nonamp_lo^ reference but longer survival relative to the CCNE1^amp_hi^ group. Both the CCNE1^nonamp_hi^ and CCNE1^amp_hi^ group express similarly high protein levels but CCNE1^amp_hi^ express higher mRNA levels than CCNE1^nonamp_hi^, suggesting that amplification‐driven CCNE1 overexpression is due to higher transcriptional activity, whereas CCNE1 overexpression in CCNE1^nonamp_hi^ cases may be more dependent on protein stabilization or lack of degradation.^32^ We, however, demonstrate that differences in *CCNE1* mRNA expression were not associated with overall survival in HGSC. We obtained consistent HRs close to 1.0 by studying 2419 samples in the current study, confirming the results from a previous OTTA study.^19^ Despite the strong correlation of mRNA with amplification status and protein levels, the lack of survival association may be caused by dilution of the mRNA signal in tumor bulk analysis with varying tumor cellularity compared with the spatially controlled CISH and IHC assays. The survival differences of the two groups with high CCNE1 protein levels still creates a conceptual dilemma because it is the protein that is exerting the function, and the mechanism of protein accumulation should not matter, unless there is a difference in the timing of expression in relation to the cell cycle or the functional quality of CCNE1 protein. In high CCNE1‐expressing breast cancer, CCNE1 can be proteolytically cleaved into low‐molecular weight derivatives.^33^ An alternative explanation might be that in the CCNE1^amp_hi^ group, other oncogenes coamplified with CCNE1 on 19q12, such as *URI* contribute to survival.^34^

Although we confirm that the group of CCNE1^amp_hi^ is associated with the shortest overall survival, we also show that this association is mainly driven by DNA copy‐number status, achieving a better stratification than protein level. However, protein level seems to provide additional information by singling out the small group of CCNE1^amp_lo^, which in the main analysis had a similar HR compared with the reference CCNE1^nonamp_lo^. In a sensitivity analysis including residual tumor, the HR was more similar to the high‐risk group CCNE1^amp_hi^. However, this was not statistically significant with the small case numbers in the CCNE1^nonamp_lo^ subgroup, and this sensitivity analysis may have introduced bias for the small subgroups that are not comparable to the overall cohort. This raises a question about the importance of the level of CCNE1 protein expression in the context of *CCNE1* high‐level amplification. Both CCNE1^amp_hi^ and CCNE1^amp_lo^ groups are similar regarding clinical parameters (i.e., age, residual disease) and rarely harbored *BRCA1/2* germline alterations; loss of RB1 was uncommon. Although this suggests no difference and assessment of the DNA copy number status would be sufficient, both groups differed in regard to the abnormal block CDKN2A expression status, which was highest in CCNE1^amp_hi^, indicating a higher RB1 pathway dependent on the CCNE1 protein level. Based on our observed differences in survival and *CCNE1* mRNA expression, together with previous study findings,^15^, ^23^ we interpret that CCNE1^amp_hi^ is different from CCNE1^amp_lo^. By focusing on the CCNE1^amp_hi^ group, IHC can be used to screen HGSC samples for CCNE1 overexpression followed by copy‐number assessment for clinical trial inclusion, which would pragmatically circumvent the limited sensitivity of CCNE1 IHC. However, the clinical significance of this relatively small group remains uncertain. We cannot entirely exclude a misclassification based on the CISH or IHC assay. Future studies should use full‐section IHC to exclude potential intratumoral heterogeneity of the protein expression and alternative copy‐number assays for the small group of CCNE1^amp_lo^ tumors. However, some *CCNE1* high‐level amplified tumors may not express high protein levels. The Cancer Genome Atlas reported that low *CDKN2A* mRNA expression is mutually exclusive with *CCNE1* amplification.^11^ We observed a small number of cases with loss of CDKN2A protein (a surrogate for *CDKN2A* deep deletions) in the CCNE1^amp_lo^ but not in the CCNE1^amp_hi^, suggesting that another concurrent RB1 pathway alteration could prevent CCNE1 protein overexpression in the context of *CCNE1* high‐level DNA amplifications.

Our results confirm that CCNE1 high‐level DNA amplifications are essentially mutually exclusive with pathogenic *BRCA1/2* germline alterations. The two exceptional cases with pathogenic *BRCA2* germline variants that were grouped as CCNE1^amp_hi^ were scored as equivocal favor high by CISH. These rare cases of “double classifiers” may require additional assays such as validated homologous recombination deficiency assays or copy number signatures to assign as homologous recombination‐deficient or homologous recombination‐proficient. From a treatment perspective, the CCNE1^amp_hi^ group had a shorter survival likely in part because of lower response to platinum‐based chemotherapy, which correlates with insensitivity to PARP inhibitors. Therefore, the CCNE1^amp_hi^ group may not respond to PARP inhibitors, making CCNE1^amp_hi^ a candidate biomarker that could be used as a negative predictive test for PARP inhibitors. This hypothesis could be tested in secondary analyses of clinical trials that include unselected HGSC patients treated with PARP inhibitors.^18^

Novel treatment approaches are required for women diagnosed with CCNE1^amp_hi^ HGSC.^35^ Bowtell and colleagues observed decreased tumorigenesis in *CDK2*‐knockout HGSC cell lines with *CCNE1* amplifications. However, the CDK2 inhibitor, dinaciclib, did not suppress tumorigenesis, probably because it is not entirely specific for CDK2.^36^ Perhaps a more specific CDK2 inhibitor could be tested on patients with HGSC and CCNE1^amp_hi^ HGSC. It remains to be seen whether redundancies in the CDK2/CCNE1 pathway (CDK1 for CDK2, CCNE2 for CCNE1) observed in normal cells pose another challenge of targeting this pathway in cancers.^3^, ^37^ In a post hoc analysis of a clinical trial investigating the Wee1 inhibitor adavosertib in combination with gemcitabine, *CCNE1*‐amplified tumors were more likely to respond.^38^ Through phosphorylation of the CDK1/CCNB complex, Wee1 kinase is an inhibitor of the G2/M transition, which is more critical for HGSC with deficient G1/S transitions. Notably, in a recent phase 2 trial, adavosertib has also shown promising response rates in CCNE1 overexpressing recurrent HGSC regardless of amplification status.^39^ Alternatively, using a CRISPR–Cas9‐screen, *PKMYT1*, which encodes a protein kinase also involved in G2/M transition, was identified as a synthetic lethal target for CCNE1 high‐expressing cells, which were sensitive to inhibition by a selective *PKMYT1* inhibitor.^40^ This suggests that perhaps both CCNE1 expression and amplification status should be assessed when testing CCNE1 as predictive marker for new molecular therapy.

Although the main function of CCNE1 is in cell‐cycle progression, the main oncogenic effect may be independent from proliferation. High proliferating HGSCs are associated with longer survival, likely because of better response to standard chemotherapy,^27^, ^28^ whereas CCNE1 alterations are associated with shorter survival and poor response to chemotherapy. CCNE1 protein expression is only weakly correlated with proliferation markers (Ki67, minichromosome maintenance complex component 3).^28^ Although uncontrolled cell‐cycle entry remains the main known function of CCNE1, overall, these data suggest that much of CCNE1's oncogenic function is related to a premature S‐phase entry resulting in chromosomal instability rather than increased proliferation.^7^, ^8^

The main limitation of our study was the assay resolution. We did not count the DNA copy number ratio by using a CEP19 control probe but focused on the presence of *CCNE1* clusters as a surrogate for high‐level amplification defined by >8 copy numbers, which was previously orthogonally validated using the NanoString CNV assay and digital polymerase chain reaction.^15^ Not using ratios prevented us from assessing low‐level gains. The prevalence of *CCNE1* high‐level amplifications is approximately half compared with previous studies reporting *CCNE1* amplification (frequency of ∼20%), which is due to the higher cutoff we used.^11^, ^12^ Our present study used CISH analysis, which is a well‐established and clinically adopted technique to interrogate genetic amplification such as evaluation of *ERBB2* amplification in breast and gastric cancer. However, next‐generation sequencing (NGS)–based assays such as whole‐genome/exome sequencing or targeted panel sequencing are being increasingly used in the clinical setting to provide more comprehensive molecular characterization of tumors, including copy number alterations. In contrast to CISH (or fluorescence in situ hybridization) assays that provide spatially focused analysis that evaluates signals only from carcinoma cells, the NGS‐based assays typically use bulk tumor samples in which tumor content can vary, and it may have lower sensitivity compared with spatially controlled assays such as in situ hybridization, particularly from samples with low tumor content in the settings of core needle biopsies or posttreatment (neoadjuvant chemotherapy) samples. Another consideration is that CISH analysis generally requires less amount of tumor tissue than NGS‐based analysis, which may be relevant in cases in which a limited amount of diagnostic tissue is available. Future studies are required to determine the clinical utility and limitations of NGS‐based assay for *CCNE1* copy number evaluation. There were limited data annotations for some analyses because of missing data for residual disease and germline *BRCA1/2* status.

In conclusion, our large‐scale validation with survival data supports the notion that CCNE1 is the most promising biomarker to define the largest subgroup of homologous recombination–proficient HGSC. CCNE1 high‐level amplifications should be studied as negative predictive markers for current standard therapies (chemotherapy, PARP inhibitors) and should be evaluated in clinical trials assessing novel treatment approaches. We propose to focus initially on the CCNE1^amp_hi^ group; CCNE1 IHC could be used as a screening tool, followed by an assessment of DNA copy number status.

## CONFLICTS OF INTEREST

The authors have no relevant conflicts of interest regarding this publication. A. Hartmann has received honoraria from BMS, MSD, Roche, AstraZeneca, Boehringer Ingelheim, AbbVie, Jansen‐Cilag, and Ipsen.

T.P. Conrads is member of the Thermo Fisher Scientific Inc scientific advisory board. P.A. Cohen is a member of the Clinic IQ Scientific Advisory Board and has received honoraria from Seqirus and Astra Zeneca. D. Bowtell has research funding from AstraZeneca, Beigene, and Genentech Roche, and is an advisor to Exo Therapeutics. U. Menon has received an honorarium from the NY Obstetrical Society and held personal shares between 1 April 2011 and 30 October 2021. T. Van Gorp has received honoraria for advisory boards from Eisai Europe (to institute), OncXerna Therapeutics (to institute), Astra Zeneca (to institute), GSK (to institute), MSD (to institute), research funding from Amgen (to institute), Roche (to institute), and AstraZeneca (to institute) and travel reimbursements from MSD, Immunogen, PharmaMar, and AstraZeneca. P. Harter has received honoraria from Amgen, AstraZeneca, GSK, Roche, Sotio, Stryker, Zai Lab, MSD, Clovis, and Eisai; has served on advisory boards for AstraZeneca, Roche, GSK, Clovis, Immunogen, MSD, and Eisai; and has received research funding (to institute) from AstraZeneca, Roche, GSK, Genmab, DFG, European Union, DKH, Immunogen, and Clovis. P. Ghatage has received honoraria from AstraZeneca, GSK, and Eisai. I. Vergote has received consulting fees from Agenus, Akesobio, AstraZeneca, Bristol‐Myers Squibb, Deciphera Pharmaceuticals, Eisai, Elevar Therapeutics, F. Hoffmann‐La Roche, Genmab, GSK, Immunogen, Jazzpharma, Karyopharm, Mersana, MSD, Novocure, Novartis, Oncoinvent, OncXerna, Sanofi, Seagen, Sotio, Verastem Oncology, and Zentalis, done contracted research with Oncoinvent AS and corporate sponsored research with Amgen and Roche, and received travel expenses/accommodations from Karyopharm and Genmab. D. Bowtell has received honoraria from AstraZeneca, has a consulting role with Exo Therapeutics Research and receives funding from Roche/Genentech, AstraZeneca and BeiGene. A. DeFazio received grant funding from AstraZeneca. A.H. had an advisory role and received honoraria from BMS, MSD, Roche, Cepheid, Qiagen, Agilent, Diaceutics, Lilly, AstraZeneca, Boehringer Ingelheim, AbbVie, Jansen‐Cilag, Pfizer, and Ipsen. R. Erber has received honoraria from Roche, Eisai, Pfizer, BioNTech, Veracyte (PROCURE), Diaceutics, and Novartis. The institution of A. Hartmann and R. Erber conducts research for AstraZeneca, Roche, Janssen‐Cilag, NanoString Technologies, Biocartis, ZytoVision, Novartis, Cepheid, and BioNTech. S. Heublein received honoraria from Clovis, Merck Sharp & Dohme Corporation and Pfizer, and has received research funding from Neuer Stiftung fuer Medizische Forschung and Novartis as well as travel support from GlaxoSmithKline.

## AUTHOR CONTRIBUTIONS

**Eun‐Young Kang:** Immunohistochemical protein and chromogen in situ hybridization scores collection and manuscript draft and revision. **Ashley Weir:** Analyses. **Kyo Farrington:** Immunohistochemical protein and chromogen in situ hybridization scores collection. **Paul D.P. Pharoah:** Statistical advice. **Susan J. Ramus:** Study conception, design, and supervision. **Martin Koebel:** Study conception, design, and supervision; immunohistochemical protein and chromogen in situ hybridization scores collection; and manuscript draft and revision. All authors contributed through collection, curation, and maintenance of respective consortia‐based, or local institution, collections of patient samples including recruitment and consenting of patients, clinical care, abstraction of clinical data, and updating of outcome and follow‐up data. All authors revised the manuscript and approved submission of the final version.

## Supporting information

Supplementary Material S1

Supplementary Material S2

Supplementary Material S3
