# Supplementary material for: CCNE1 and survival of patients with tubo‐ovarian high‐grade serous carcinoma: An Ovarian Tumor Tissue Analysis consortium study
Source: Cancer. 2022 Dec 26;129(5):697–713. doi: 10.1002/cncr.34582 (PMC10107112; doi:10.1002/cncr.34582)
Supplement: Supplementary file 2 — Supplementary Material S2 [file CNCR-129-697-s002.docx]

| **Table S2 Sensitivity analyses including residual disease as covariate:** Multivariate association between the expression and amplification of CCNE1 and overall survival in high-grade serous carcinoma (n=2026) | | | | | |
| --- | --- | --- | --- | --- | --- |
| **CCNE1 profile** | **n**${}^{a}$ | **5-yr survival (% ± SE)** | **Hazard ratio (95% CI)**${}^{b}$ | **p-value** | |
| *CCNE1* CISH score 0 | 1613 | 42.3 ± 1.3 | ref | 0.0744 | |
| *CCNE1* CISH score 1 | 200 | 41.7 ± 3.6 | 0.99 (0.83-1.19) |  | |
| *CCNE1* CISH score 2 | 39 | 32.0 ± 7.9 | 0.89 (0.61-1.29) |  | |
| *CCNE1* CISH score 3 | 174 | 30.3 ± 3.7 | **1.27 (1.06-1.52)*** |  | |
| CCNE1 IHC score 0 | 1187 | 42.2 ± 1.5 | ref | **0.0374*** | |
| CCNE1 IHC score 1 | 206 | 45.4 ± 3.7 | 0.98 (0.82-1.18) |  | |
| CCNE1 IHC score 2 | 180 | 42.3 ± 3.8 | 0.95 (0.79-1.16) |  | |
| CCNE1 IHC score 3 | 453 | 35.7 ± 2.4 | **1.20 (1.05-1.37)*** |  | |
| CCNE1^nonamp_lo^ | 1364 | 42.9 ± 1.4 | ref | 0.137 | |
| CCNE1^nonamp_hi^ | 449 | 40.3 ± 2.4 | 1.09 (0.96-1.25) |  | |
| CCNE1^amp_lo^ | 29 | 28.0 ± 8.9 | 1.27 (0.81-1.99) |  | |
| CCNE1^amp_high^ | 184 | 30.9 ± 3.6 | **1.20 (1.00-1.43)*** |  |  |
| ${}^{a}$ The same cohort was assessed in univariate survival analysis  ${}^{b}$ Hazard ratio adjusted for patient age, stage and residual disease, with stratification by OTTA study; Cox proportional regression modelling was used to calculate p-values and define significance. Statistically significant values shown in bold; * p<0.05  Cyclin E1 (CCNE1); High-grade serous ovarian carcinoma (HGSC); Overall survival (OS); Standard error (SE); Hazard ratio (HR); Confidence interval (CI); Chromogenic in situ hybridization (CISH); Immunohistochemistry (IHC); negative for CCNE1 high-level amplification (CCNE1^nonamp^); CCNE1 high-level amplification (CCNE1^amp^); negative for CCNE1 protein overexpression by immunohistochemistry (CCNE1^lo^); CCNE1 protein overexpression by immunohistochemistry (CCNE1^hi^) | | | | | |

**Table S3** Binarized CCNE1 DNA amplification and protein level combinations

| **CCNE1 IHC,**  **n (%)** | ***CCNE1* CISH** | | | | **p-value** |
| --- | --- | --- | --- | --- | --- |
|  |  | **0-1** | **2-3** | **Total** | **<0.0001*** |
|  | **0-1** | 2064 (76.4) | 61 (18.7) | 2125 (70.2) |  |
|  | **2-3** | 639 (23.6) | 265 (81.3) | 904 (29.8) |  |
|  | **Total** | 2703 (89.2) | 326 (10.8) | 3029 (100.0) |  |
| * Chi-squared testing was used to assess significant differences in binarized ISH and IHC CCNE1 scores in HGSC. Statistically significant values shown in bold; *p<0.05  **Cyclin E1 (CCNE1); Chromogenic *in situ* hybridization (CISH); Immunohistochemistry (IHC)** | | | | | |

**Table S4** Univariate associations of the combined CCNE1 groups with type of primary chemotherapy regimen.

|  | **CCNE1 combined groups** | | | |  |  |
| --- | --- | --- | --- | --- | --- | --- |
| **Primary Treatment** | CCNE1^nonamp_lo^ | CCNE1^amp_lo^ | CCNE1^nonamp_hi^ | CCNE1^amp_high^ | **Total** | **p-value*** |
| PDS | 851 (69.0) | 41 (3.3) | 239 (19.4) | 103 (8.4) | 1234 | 0.1649 |
| NACT | 66 (80.5) | 1 (1.2) | 10 (12.2) | 5 (6.1) | 82 |  |
| Unknown | 749 | 11 | 211 | 101 | 1072 |  |
| **Total** | **1666** | **53** | **460** | **209** | **2388** |  |

* Chi-squared testing was used to calculate p-values. Statistically significant values, where p<0.05, have been shown in bold. This does not include cases where mutational status was unknown.

Percentages of row total given in brackets.

Cyclin E1 (CCNE1); High-grade serous ovarian carcinoma (HGSC); primary debulking surgery (PDS); neoadjuvant chemotherapy (NACT); negative for CCNE1 high-level amplification (CCNE1^nonamp^); CCNE1 high-level amplification (CCNE1^amp^); negative for CCNE1 protein overexpression by immunohistochemistry (CCNE1^lo^); CCNE1 protein overexpression by immunohistochemistry (CCNE1^hi^)

| **Table S5** Clinicopathological parameters of mRNA expression/NanoString 2 cohort (n=2419) | | | | |
| --- | --- | --- | --- | --- |
| **Clinicopathological variable** | **NanoString 2 Cohort** | | | **p-value**${}^{a}$ |
|  | **Top 10% expression** | **Remaining** | **Total** |  |
| **Number of cases,**  **n (%)**${}^{\boldsymbol{b}}$ | 242 (10.0) | 2177 (90.0) | 2419 (100.0) |  |
| **Age at diagnosis, years** |  |  |  |  |
| Mean ± SD | 66.1±9.3 | 61.5±10.9 | 62.0 ±10.9 |  |
| Median | 66 | 62 | 63 |  |
| Range | 39-90 | 29-93 | 29-93 |  |
| **Stage, n (%)**${}^{\boldsymbol{c}}$ |  |  |  | 0.0638 |
| FIGO I, II (local/regional), n (%) | 42 (17.5) | 283 (13.2) | 325 (13.6) |  |
| FIGO III, IV (distant), n (%) | 198 (82.5) | 1865 (86.8) | 2063 (86.4) |  |
| Unknown${}^{d}$ | 2 | 29 | 31 |  |
| **Residual disease**${}^{c}$ |  |  |  | 0.8881 |
| Absent, n (%) | 56 (47.5) | 596 (46.8) | 652 (46.8) |  |
| Present, n (%) | 62 (52.5) | 678 (53.2) | 740 (53.2) |  |
| Unknown${}^{d}$ | 124 | 903 | 1027 |  |
| ${}^{a}$ Chi-squared testing was used to calculate p-values. Statistically significant values shown in bold; *p<0.05.  ${}^{b}$The proportion of cases in each score stratum is given as a percentage of the total patients examined  ${}^{c}$The proportion of cases is given as a percentage of the total cases within each NanoString 2 cohort  ${}^{d}$ Cases of unknown status were not included in the calculation of the Chi-squared statistic  **Standard deviation (SD); International Federation of Gynecology and Obstetrics (FIGO)** | | | | |

**Table S6** Multivariable survival analysis of *CCNE1* mRNA expression

| Study |  | Gene | Analysis | N | Hazard ratio | 95% CI | | p-value | Inclusion criteria |
| --- | --- | --- | --- | --- | --- | --- | --- | --- | --- |
| NanoString study 1 | Ref Millstein et al. | *CCNE1* | per 1 SD change expression | 3769 | 1.03 | 0.98 | 1.07 | 0.22 | PDS |
| NanoString study 1 | Ref Millstein et al. | *CCNE1* | per 1 SD change expression | 3387 | 1.04 | 0.99 | 1.08 | 0.08 | PDS ovary |
| NanoString study 2 | Current study | *CCNE1* | per 1 SD change expression | 2419 | 1.00 | 0.94 | 1.06 | 0.96 | all samples |
| NanoString study 2 | Current study | *CCNE1* | per 1 SD change expression | 1840 | 1.01 | 0.95 | 1.08 | 0.68 | PDS |
| NanoString study 2 | Current study | *CCNE1* | per 1 SD change expression | 1436 | 0.99 | 0.92 | 1.07 | 0.85 | PDS ovary |
| NanoString study 2 | Current study | *CCNE1* | per 1 SD change expression | 515 | 0.99 | 0.87 | 1.13 | 0.93 | NACT |
| NanoString study 2 | Current study | *CCNE1* | per 1 SD change expression | 362 | 0.97 | 0.83 | 1.12 | 0.66 | NACT ovary |
| NanoString study 2 | Current study | *CCNE1* | top 10% vs rest | 2419 | 1.06 | 0.88 | 1.27 | 0.53 | all samples |
| NanoString study 2 | Current study | *CCNE1* | top 10% vs rest | 1840 | 1.08 | 0.88 | 1.34 | 0.46 | PDS |
| NanoString study 2 | Current study | *CCNE1* | top 10% vs rest | 1436 | 1.02 | 0.80 | 1.30 | 0.89 | PDS ovary |
| NanoString study 2 | Current study | *CCNE1* | top 10% vs rest | 515 | 1.04 | 0.70 | 1.56 | 0.83 | NACT |
| NanoString study 2 | Current study | *CCNE1* | top 10% vs rest | 362 | 0.76 | 0.46 | 1.24 | 0.26 | NACT ovary |

Hazard ratio adjusted for patient age and stage, with stratification by OTTA study; Cox proportional regression modelling was used to calculate p-values and define significance.

Inclusion criteria: Analysis were stratified based on samples selected for timing of chemotherapy and anatomical site of the specimen: PDS – primary debulking surgery represent chemo naïve samples, NACT – post neoadjuvant chemotherapy represent samples exposed to at least 3 cycles of platinum-based chemotherapy; ovary – means the tumor specimen was sampled from the ovarian tumor excluding other samples from the omentum etc.
