# Supplementary material for: CCNE1 and survival of patients with tubo‐ovarian high‐grade serous carcinoma: An Ovarian Tumor Tissue Analysis consortium study
Source: Cancer. 2022 Dec 26;129(5):697–713. doi: 10.1002/cncr.34582 (PMC10107112; doi:10.1002/cncr.34582)
Supplement: Supplementary file 3 — Supplementary Material S3 [file CNCR-129-697-s003.pdf]

Studes contributing t	site	Name	Location	Years	Ascertainment of patients and clinical data	Pathology data and review	Ethics committee	Informed consent
1	AOC	Australian Ovarian Cancer Study	Australia	2002-2006	Treatment centers throughout Australia; cancer registries serving Queensland, South and West Australia; regular follow- up by medical record review	Central review of pathology reports and histological slides by study pathologist	Peter MacCallum Cancer Centre Human Research Ethics Committee	Yes
2	BAV	Bavarian Ovarian Cancer Study	Germany	2002-2006	Gynaecologic Oncology Center at the Comprehensive Cancer Center Erlangen-Nuremberg	Central review of pathology reports and histological slides by study pathologist	Ethics Committee of the Friedrich-Alexander-University Erlangen-Nuremberg	Yes
3	BGS	Breaktfhrough Generations Study	UK	1988-2013	Follow-up of a national general population cohort study	Pathology reports	Ethics Committee is South East Multi-Centre Research Ethics Committee	Yes
4	BRZ	Brazil Gynecologic Tumor Bank (BRZ) study	Brazil	1987-2010	University Hospital of Ribeirao Preto School of Medicine (HCRP), case series with prospective follow up	Pathology reports and histologic slides reviewed by HCRP gynecologic pathologists	Research Ethics Committee of Hospital das Clínicas of the Ribeirão Preto Medical School	No / pathology material
5	CAL	Calgary Serous Carcinoma Study	Canada	2003-2007	Hospital based retrospective observational study	Central review of pathology reports and histological slides by study pathologist	Health Research Ethics Board of Alberta	No / pathology material
6	CNI	CNIO Ovarian Cancer Study	Spain	2006-2013	Hospitals in Madrid in Medical Oncology Divisions	Pathology information was obtained through medical chart review in the Medical Oncology units	Bioethics and Animal Welfare Committee of the Carlos III Health Institute	Yes
7	DUK	Duke University Medical Center	United States	2008-2009	Duke University Medical Center, North Carolina	Stained histological slides reviewed centrally by study pathologist	Duke University Health System Institutional Review Board	Yes
8	HAW	Hawaii Ovarian Cancer Study	US	1993-2008	Hawaii Tumor Registry and medical records	Central review of pathology reports and histological slides by study pathologist	University of Hawaii, Committee on Human Studies	Yes
9	HMC	(HM-Ciocc) HM Hospitales – Centro Integral Oncológico HM Clara Campa	Spain	2016-2021	Department of Gynecologycal Tumors at HM Hospitals	Central review of pathology reports and histological slides by study pathologist	Research and Ethics committee of HM hospital	Yes
10	HOP	Hormones and Ovarian Cancer PrEdiction	United States	2003-2009	Hospital registries and active surveillance of medical practices in Western PA	Pathology information was obtained through medical chart review in the Medical Oncology units	University of Pittsburgh Insititutional Review Board	Yes
11	KMC	Biospecimen Repository Core Facility - The University of Kansas Medical Center	United States	2010 - 2016	Tumor registry and theUniversity of Kansas Hospital medical records	Pathology reports and histologic slides reviewed by gynecologic pathologist	Institutional Review Board of the University of Kansas Medical Center	Yes
12	LAX	Women's Cancer Research Program - Cedars-Sinai Medical Center	United States	1989-present	Women's Cancer Program Biorepository	Central review of pathology reports and histological slides by study pathologist	Institutional Review Board 3 of Cedars-Sinai Medical Center	Yes
13	MAY	Mayo Clinic Ovarian Cancer Study	United States	2000-2013	Mayo Clinic medical records and death certificates	Central review of pathology reports and histological slides by study pathologist	Institutional Review Board of Mayo Clinic	Yes
14	NCT	Natl. Centre of Tumor Diseases and the Dept. of Pathology, Heidelberg	Germany	2004-2016	Natl. Centre of Tumor Diseases and the Dept. of Pathology, Heidelberg	Pathology reports and histologic slides reviewed by gynecologic pathologist	Ethics-Committee of the Medical Faculty at the University of Heidelberg	Yes
15	POC	Polish Ovarian Cancer Study	Poland	2000-2003	Hospital records and cancer registries serving Warsaw and Lodz	Stained histological slides reviewed centrally by study pathologist	Bioethical Committee of Pomeranian Medical University	Yes
16	SEA	Study of Epidemiology and Risk Factors in Cancer Heredity	UK	1998-present	Eastern Region Cancer Intelligence Unit, West Midlands Cancer Intelligence Unit, and multiple cancer networks	Central review of pathology reports and histological slides by study pathologist	Cambridgeshire 4 Research Ethics Committee	Yes
17	TUE	Tuebingen University Women's Hospital (TUE) study	Germany	1999-2008	Department of Obstetrics and Gynaecology, Eberhard Karls Universitats Tübingen, Tübingen Germany	Pathology reports and histologic slides reviewed by gynecologic pathologist	Ethics-Committee at the Medical Faculty and at the University Hospital of Tübingen	Yes
18	UKO	United Kingdom Ovarian Cancer Population study	UK	2006-2010	Ten major Gynecologic Oncology NHS centers in England, Wales and Northern Ireland; cancer registries; NHS Information Centre for Health and Social Care (England and Wales) and Central Services Agency (Northern Ireland)	Central review of pathology reports by gynaecologic oncologist	National Health Service Central Office for Research Ethics Committees (COREC) and The Joint University College London/University College London Hospital Committee on the Ethics of Human Research (Committee A)	Yes
19	VAN	Vancouver Ovarian Cancer Study	Canada	1984-2000	Ovarian Cancer Registry serving British Columbia and the Cheryl Brown Outcomes Unit	Central review of pathology reports and histological slides by study pathologist	University of British Columbia - British Columbia Cancer Agency Research Ethics Board	Some cases Yes and some cases No / pathology material
20	WMH	WestMead Hospital	Australia	1992-present	The Crown Princess Mary Cancer Centre and affiliated hospitals	Pathology reports and diagnostic slides reviewed by panel of gynecologic pathologists	Western Sydney Local Health District, Human Research Ethics Committee	Yes

Studies contributing for mRNA only

1	BEL	ı, Department of Gynaecological Oncolo	Belgium	2007-2017	University Hospitals Leuven (UZL), departm	Pathology reports and histologic slides reviewe	Ethics Committee of the University Hospitals Leuven	Yes
2	COE	issue and Data Acquisition Network Stu	United States	2012-2021	GYN-COE Biobank	Central review of pathology reports and histological slides by study pathologist	Western IRB	Yes
3	COH	COHBRI Ovarian Study	United States	2012-2021	City of Hope Medical Center	Central review of pathology reports and histological slides by study pathologist	City of Hope Institutional Review Board	Yes
4	HSA	Health Science Alliance Biobank	Australia	2012-present	Prince of Wales Hospital, Royal Hospital for Women, St George Hospital, Sydney NSW. Survival data Australian Institute of Health and Welfare	Pathology reports and histological slides reviewed by study pathologist	South Eastern Sydney Local Health District Human Research Ethics Committee	Yes
5	JGO	Japanese gynecologic oncology project	Japan	2008-2018	Keio Women's Health Biobank, Department of Obstetrics & Gynecology, Keio University School of Medicine	Pathology reports and histological slides reviewed by study pathologist	Keio University School of Medicine Ethics Committee.	Yes
6	KEM	Kliniken Essen-Mitte, Department for Gynecology and Gynecologic Oncology	Germany	2010-2018	Hospital based prospectively maintained, retrospective registry	Pathology reports and histological slides reviewed by study pathologist	Ethical Reviewboard Landesärztekammer Nordrhein	No
7	NEC	New England Case Control Study	United States	1992- 2008	Hospital tumor boards and cancer registries; (	Pathology reports and histologic slides reviewe	Mass General Brigham Institutional Review Board	Yes
8	NTH	r institute - Netherlands comprehensive c	Netherlands	2008-2015	The Netherlands cancer registry	Central review of pathology reports and histological slides by two independend gynecologic pathologists	Institutional Review Board of the Netherlands cancer registry and the institutional Review Board of the Netherlands cancer institute	No
9	PVD	Pelvic Mass	Danish	2004-present	Hospital based prospektive study	Pathology reports	De Videnskabsetiske Komiteer for Region Hovedstaden	Yes
10	SWE	Sweden Western Region Ovarian Cancer Study	Sweden	2001-2016	Sahlgrenska University Hosptial, medical records and the clinical cancer register in the western Sweden health care region and Swedish death register	Original pathology report. For specific studies i.e. Tissue-micro-arrays re-evaluated by gyno-pathologist	Swedish Ethical Review Authority	Yes
11	TVB	OVAL BC	Canada	2001 and 2012	Province of British Columbia	IHC supported slide review by gynecological p	University of British Columbia - British Columbia Cancer Agency Research Ethics Board	Some cases Yes and some cases No / pathology material
12	USC	Los Angeles County Case-Control Study of Ovarian Cancer	US	1992-2008	Los Angeles County SEER Program	Pathology reports and histological slides reviev	Institutional Review Board Health Research Association and Institutional Review Board University of Southern California School of Medicine	Yes
13	WAGO	Western Australia Gynaecological Oncology	Australia	2013 - present	St John of God Hospital, Subiaco, WA and King Edward Memorial Hospital, Perth WA	Pathology reports and histological slides reviewed by study pathologist	St John of God Healthcare Human Research Ethics Committee and the Women and Newborn Human Research Ethics Committee	Yes
